# Synthesis of novel 13α-estrone derivatives by Sonogashira coupling as potential 17β-HSD1 inhibitors

**DOI:** 10.3762/bjoc.13.126

**Published:** 2017-06-30

**Authors:** Ildikó Bacsa, Rebeka Jójárt, János Wölfling, Gyula Schneider, Bianka Edina Herman, Mihály Szécsi, Erzsébet Mernyák

**Affiliations:** 1 Department of Organic Chemistry, University of Szeged, Dóm tér 8, H-6720 Szeged, Hungary; 2 1st Department of Medicine, University of Szeged, Korányi fasor 8–10, H-6720 Szeged, Hungary

**Keywords:** benzofuran, 13α-estrone, 17β-HSD1 inhibition, partial saturation, Sonogashira coupling

## Abstract

Novel 13α-estrone derivatives were synthesized by Sonogashira coupling. Transformations of 2- or 4-iodo regioisomers of 13α-estrone and its 3-methyl ether were carried out under different conditions in a microwave reactor. The 2-iodo isomers were reacted with *para*-substituted phenylacetylenes using Pd(PPh_3_)_4_ as catalyst and CuI as a cocatalyst. Coupling reactions of 4-iodo derivatives could be achieved by changing the catalyst to Pd(PPh_3_)_2_Cl_2_. The product phenethynyl derivatives were partially or fully saturated. Compounds bearing a phenolic OH group furnished benzofurans under the conditions used for the partial saturation. The inhibitory effects of the compounds on human placental 17β-hydroxysteroid dehydrogenase type 1 isozyme (17β-HSD1) were investigated by an in vitro radiosubstrate incubation method. Certain 3-hydroxy-2-phenethynyl or -phenethyl derivatives proved to be potent 17β-HSD1 inhibitors, displaying submicromolar IC_50_ values.

## Introduction

Synthetic modifications of the naturally occurring female prehormone estrone may lead to compounds with diverse biological activities, for example with antitumor effect [1]. One of the main requirements of estrone anticancer derivatives is the lack of their hormonal activity. Several core-modified estrones have recently been produced and diversified in order to get selectively acting compounds [2–4]. One opportunity for that is the inversion of the configuration at C-13, which is accompanied by drastic conformational change for the overall molecule resulting from the *cis* junction of rings C and D [2]. The influence of inversion of the configuration at C-13 in 3,17-estradiols on their in vivo and in vitro estrogenic activity was shown by Poirier et al. [5]. They demonstrated that 13 epimers exhibit no substantial binding affinity for the estrogen receptor alpha and no uterotropic activity. Accordingly, the 13α-estrane core may serve as fundamental moiety for the design of hormonally inactive estrone derivatives bearing promising biological activities. We recently published the syntheses and the in vitro biological evaluations of several 13α-estrone derivatives [6–9]. Certain compounds proved to be biologically active, bearing substantial antiproliferative or enzyme inhibitory potential [7–8]. Most literature data are mainly about 13α-estrones substituted in ring D, but compounds modified in ring A are rarely described [10–11]. More recently we have disclosed ring A halogenations in this series [12]. Electrophilic brominations or iodinations were carried out, furnishing 2-, 4- or 2,4-bis-halogenated compounds. All the halogenated 3-hydroxy and the 4-substituted regioisomers of 3-methyl ethers displayed substantial inhibitory activity against the 17β-hydroxysteroid dehydrogenase type 1 enzyme (17β-HSD1). Certain derivatives displayed a similar or more pronounced effect than those of their parent compounds 13α-estrone or 13α-estrone 3-methyl ether [13]. The 17β-HSD1 enzyme is responsible for the stereospecific reduction of prehormone estrone into the main estrogenic hormone 17β-estradiol [14–15]. 17β-Estradiol may enhance the proliferation of certain cancer cells [16]. The inhibition of 17β-HSD1 provokes an antitumor effect in hormone dependent cancers, hence 17β-HSD1 inhibitors could have good prospects as anti-estrogen therapeutics [17–18]. The recently synthesized halogenated 13α-estrones, in addition to their pharmacological importance, may serve as appropriate starting compounds for Pd-catalyzed C–C coupling reactions. Some Sonogashira couplings on estrane, but not on the 13α-estrane core have been performed at C-2, -3, -11, -16 and -17. To the best of our knowledge, 4-coupled regioisomers have not been synthesized to date [19]. Couplings of steroidal alkynes with small molecular halides are already described, and reactions of steroidal halides or triflates with small molecular alkynes also exist [20]. Certain phenethynyl estrone derivatives described in the literature possess substantial biological activities. Möller et al. performed the couplings of 2-iodoestrone-3-acetate with phenylacetylene using Pd(OAc)_2_ and CuI as catalysts [21]. They did not investigate the influence of the nature of the substituent on the phenyl ring of the acetylene on the course of the reactions. They carried out the full saturation of the C≡C bond of the 2-phenethynyl estrone with palladium on charcoal, furnishing the 2-phenethyl-substituted derivative. However, they did not study the partial saturation of the estrone alkyne moiety. The 2-phenethyl and 2-phenethynyl derivatives proved to be potent 17β-HSD1 inhibitors with the fully-saturated compound being slightly more potent.

The aim of the present study was to develop facile and effective Sonogashira coupling methods for the preparation of 2- or 4-phenethynyl derivatives in the 13α-estrone series. 2- or 4-iodo-13α-estrone and their 3-methyl ethers were chosen as starting compounds. The partial or full saturation of the C≡C bond of certain 2- or 4-regioisomeric phenethynyl compounds was also planned. We intended to investigate the potential inhibitory effects of the novel 13α-estrones toward human placental 17β-HSD1 activity in vitro.

## Results and Discussion

### Synthetic work

#### Sonogashira coupling

**Scheme 1:**
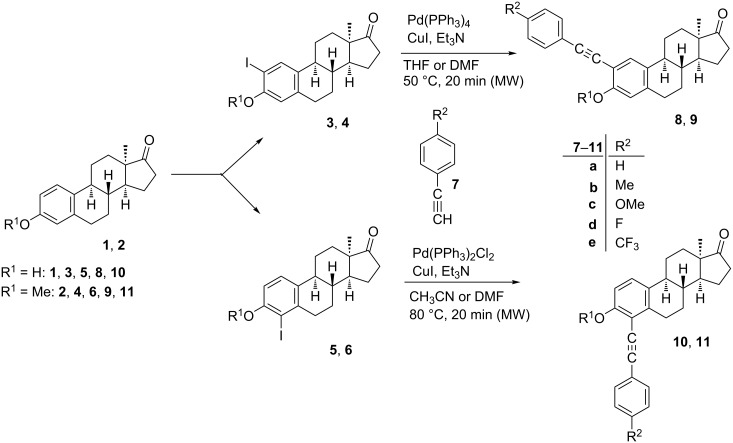
Syntheses of 2- or 4-phenethynyl-13α-estrones (**8**–**11**) by Sonogashira coupling.

Iodo compounds **3**–**6** synthesized recently have been chosen as starting materials for the Sonogashira couplings, since the reactivity of the aryl iodides is higher than that of their bromo counterparts (Scheme 1) [22]. The optimizations of the coupling reactions were carried out using phenylacetylene (**7a**) as a model reagent. The optimal reaction conditions were found to differ depending on the position of the iodo substituent on the sterane skeleton (Scheme 1). Couplings at C-2 could efficiently be achieved using 0.1 equiv of Pd(PPh_3_)_4_ and CuI in tetrahydrofuran (THF) or dimethylformamide (DMF) as solvent in the presence of Et_3_N as a base at 50 °C for 20 min in a microwave reactor. 4-Phenylalkynyl regioisomers (**10a**, **11a**) were obtained in high yields using 0.05 equiv of Pd(PPh_3_)_2_Cl_2_ and CuI in CH_3_CN or DMF, in the presence of Et_3_N as a base at 80 °C for 20 min in a microwave reactor. After establishing the most favorable reaction conditions, the Sonogashira reactions (of both regioisomers) were carried out with several *para*-substituted phenylacetylenes (**7b**–**e**). All the couplings resulted in the desired products (**8**–**11**) in high yields. The newly synthesized 4-phenethynyl derivatives are the first 4-substituted Sonogashira coupled estrones in the literature. The structures of the new compounds were confirmed by ^1^H, ^13^C and two-dimensional NMR measurements (see Supporting Information File 1).

#### Full and partial saturation of the alkyne moiety

**Scheme 2:**
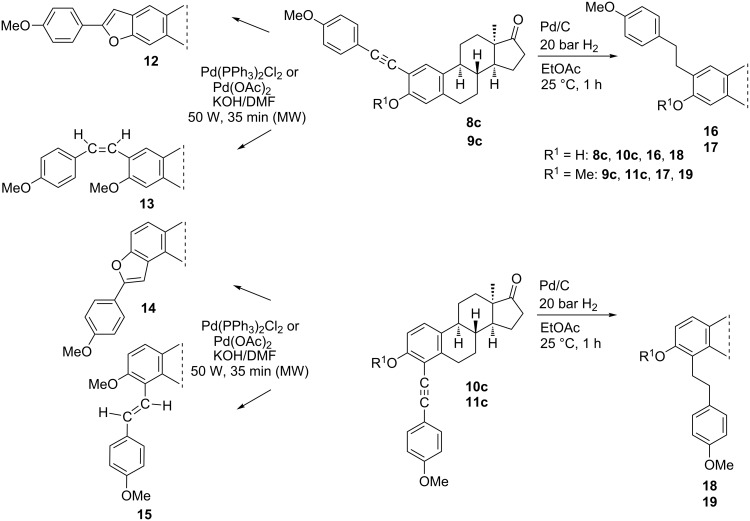
Partial or full hydrogenation of compounds **8c**–**11c**.

We have chosen four 4”-methoxy-substituted phenylalkynyl compounds (**8c**–**11c**) for partial or full saturation of the C≡C bond in both the 3-OH and the 3-OMe series (Scheme 2). The *trans* counterpart of the resulting diphenylethenyl moiety is related to the fully-methoxylated derivative of resveratrol (3,5,4’-trihydroxystilbene), a compound exhibiting diverse biological activities [23–24]. The chemo- and stereoselective semihydrogenation of internal alkynes may be achieved by two main catalytic methods: with molecular hydrogen using Lindlar’s catalyst [25–26] or by transfer hydrogenation with hydrogen donors [27–28]. Additionally, alkynes undergo reduction with diimide to produce *cis*-alkenes [29]. Li et al. carried out the semihydrogenation of different arylacetylenes using Pd(OAc)_2_ or Pd(PPh_3_)_2_Cl_2_ as the catalyst and DMF/KOH as a hydrogen source, under conventional heating [30]. The first catalyst afforded *cis*-alkenes in high yields with excellent chemo- and stereoselectivity. The latter catalyst displayed lower catalytic activity and stereoselectivity. The stereoselectivity of the semihydrogenation process may play a crucial role concerning the biological activity of the resulting alkenes, since geometrical isomers may possess different biological functions [31]. Here we performed the partial saturation of compounds **8c**–**11c** by the modified procedure of Li et al. using Pd(OAc)_2_ or Pd(PPh_3_)_2_Cl_2_ as a catalyst, and DMF/KOH as a hydrogen source, in a microwave reactor. The *cis*-alkene **13** and the *trans*-alkene **15** were formed chemo- and stereoselectively under the applied conditions. The different stereochemical outcome of the hydrogenations of the two regioisomers presumably arose from the steric hindrance caused by the vicinity of ring B in the case of compound **15**.

The *cis* or *trans* orientation of the resulting geometric isomers was deduced from the vicinal coupling constants according to the literature data, because *cis* and *trans* couplings across a double bond are very reliable indicators of stereochemistry [32–33]. In the case of the 2-regioisomer **13**, the signals of the vicinal olefinic protons appear as a singlet with double intensity, similar to those of 2,4’-dimethoxystilbene [32–33]. In the ^1^H NMR spectrum of the 4-substituted counterpart **15**, the olefinic protons are shown as doublets with a large coupling constant of 12.2 Hz, which refers to their *trans* arrangement. Under the conditions used for the partial saturation, the ethynyl derivatives bearing a phenolic OH group (**8c**, **10c**) furnished benzo[*b*]furans **12** and **14**. There are literature reports about similar transition-metal-catalyzed cyclizations of *o*-alkynylphenols to construct benzofurans [34–35]. These heterocycles are important structural units in a variety of biologically active natural or synthetic compounds [36–37]. Full hydrogenation of the 2- or 4-phenethynyl intermediates (**8c**–**11c**) with palladium-on-charcoal furnished the 2- or 4-phenethyl-substituted derivatives (**16**–**19**).

### In vitro 17β-HSD1 enzyme inhibition test

With the new compounds in hand (**8**–**19**, Table 1), we also determined their in vitro inhibitory potencies on human placental 17β-HSD1. In the 3-OH series, all the 2-phenylalkynyl regioisomers **8a**–**e** proved to be effective inhibitors with IC_50_ values depending on the nature of the 4”-functional group. The most potent compound was unsubstituted **8a** with an IC_50_ of 0.15 μM. The 4-substituted regioisomers **10a**–**e** inhibited the enzyme scarcely, suppressing the conversion by less than 15%. The phenylalkynyl derivatives in the 3-OMe series **9a**–**e** and **11a**–**e** exerted weak inhibitions. Phenylalkenyl compounds **13** and **15** and benzofuran compounds **12** and **14** displayed weaker inhibitory activity than their alkynyl counterparts **8c** and **10c**. The full saturation (leading to compounds **16**–**19**) did not influence the inhibitory potential markedly. The weak inhibitory activities of **9c**, **10c** or **11c** were not improved in compounds **17**, **18** or **19**, whereas the good inhibitory effect of the 2-regioisomer **8c** was retained in compound **16**.

**Table 1:** 17β-HSD1 inhibition data of Sonogashira coupled compounds and their precursors (**1**–**6**) [12–13] indicated with an asterisk (*).

Structure	Compound	R^1^	R^2^	Relative conversion^a^ ± SD (%) or IC_50_ ± SD (μM)

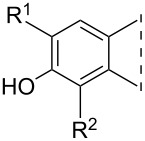	**1**	H	H	IC_50_ = 1.2*
	**3**	I	H	IC_50_ = 0.59*
	**5**	H	I	IC_50_ = 1.0*

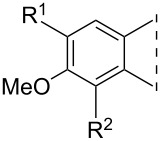	**2**	H	H	IC_50_ = 5.5*
	**4**	I	H	IC_50_ > 10*
	**6**	H	I	IC_50_ = 0.56*

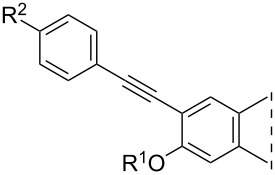	**8a**	H	H	IC_50_ = 0.15 ± 0.02
	**8b**		Me	IC_50_ = 1.40 ± 0.78
	**8c**		OMe	IC_50_ = 0.23 ± 0.03
	**8d**		F	IC_50_ = 0.30 ± 0.08
	**8e**		CF_3_	IC_50_ = 0.93 ± 0.13
	**9a**	Me	H	88 ± 12
	**9b**		Me	84 ± 5
	**9c**		OMe	85 ± 1
	**9d**		F	94 ± 5
	**9e**		CF_3_	76 ± 1

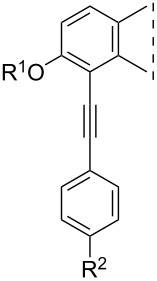	**10a**	H	H	92 ± 15
	**10b**		Me	89 ± 0.4
	**10c**		OMe	91 ± 2
	**10d**		F	96 ± 7
	**10e**		CF_3_	85 ± 1
	**11a**	Me	H	92 ± 12
	**11b**		Me	52 ± 12
	**11c**		OMe	83 ± 8
	**11d**		F	83 ± 1
	**11e**		CF_3_	79 ± 3

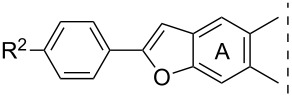	**12**	–	OMe	92 ± 2
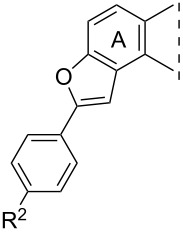	**14**			102 ± 6

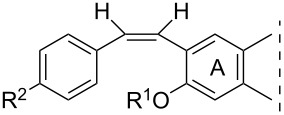	**13**	Me	OMe	70 ± 6
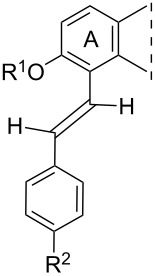	**15**			80 ± 12

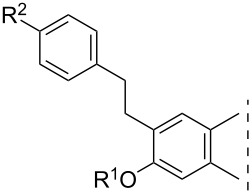	**16**	H	OMe	IC_50_ = 0.47 ± 0.04
	**17**	Me		63 ± 8

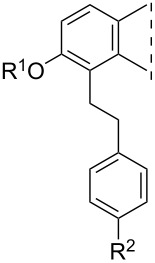	**18**	H	OMe	98 ± 3
	**19**	Me		94 ± 1

^a^At 10 μM, non-inhibited control 100%. Reference for precursors (**1**–**6**) [12–13].

When all the inhibition data of the novel compounds and their precursors from Table 1 are taken into consideration, some valuable structure–activity relationships appear. 13α-Estrone (**1**) displays 17β-HSD1 inhibitory potential similar to that of the natural substrate estrone. Iodination at C-2 of **1** improves the inhibitory potential, resulting in a submicromolar IC_50_ for compound **3**. Phenylalkynylation of the 2-iodo compound **3** retains or further improves the inhibition, depending on the nature of the substituent at C-4”. Concerning the 4-regioisomers, iodination leads to an efficiency similar to that of compound **1**, whereas the inhibition is lost following C–C coupling. 13α-Estrone 3-methyl ether **2** possesses a weaker inhibitory effect than the 3-hydroxy compound **1**. Iodination or phenylalkynylation at C-2 diminishes inhibition of **2**. Introducing iodine onto C-4 of compound **2** leads to a 10-fold decrease in its IC_50_ value. 4-Phenylalkynyl derivatives **10** and **11**, nevertheless, exert weak inhibitions on the estrone to 17β-estradiol conversion

The results reveal a great influence of the 2,4-regioisomerism on the inhibition potential of the iodinated 3-methyl ethers **4** and **6**, the phenylalkynyl **8** and **10** and the phenylalkyl **16** and **18** 3-hydroxy compounds.

## Conclusion

In conclusion, we described here an efficient synthetic microwave procedure for the synthesis of novel phenylalkynyl derivatives of 13α-estrone (**1**) and its 3-methyl ether **2**. The steroidal alkynes were chemo- and stereoselectively hydrogenated by transfer hydrogenation in a microwave reactor, furnishing alkenes or benzofurans depending on the nature of the substituent at C-3. Full hydrogenations of certain phenethynyl derivatives were also achieved. The newly-synthesized potent 17β-HSD1 inhibitors may serve as suitable tools for ligand-based enzyme studies. Further derivatizations of our compounds may provide promising candidates for drug development in order to get nanomolar inhibitors.

## Supporting Information

File 1 Experimental procedures for compounds **8**–**19** and their ^1^H, ^13^C NMR, MS, elemental analysis data.
