# Supplementary material for: Synthesis of novel 13α-estrone derivatives by Sonogashira coupling as potential 17β-HSD1 inhibitors
Source: Beilstein J Org Chem. 2017 Jun 30;13:1303–9. doi: 10.3762/bjoc.13.126 (PMC5496578; doi:10.3762/bjoc.13.126)
Supplement: File 1 — Experimental procedures for compounds 8–19 and their 1H, 13C NMR, MS, elemental analysis data. [file Beilstein_J_Org_Chem-13-1303-s001.pdf]

Supporting Information

for

Synthesis of novel 13α-estrone derivatives by Sonogashira coupling as potential 17β-HSD1 inhibitors

Ildikó Bacsa[1], Rebeka Jójárt[1], János Wölfling[1], Gyula Schneider[1], Bianka Edina Herman[2], Mihály Szécsi[2] and Erzsébet Mernyák*[1]

Address: [1]Department of Organic Chemistry, University of Szeged, Dóm tér 8, H-6720 Szeged, Hungary and [2]1st Department of Medicine, University of Szeged, Korányi fasor 8–10, H-6720 Szeged, Hungary

Email: Erzsébet Mernyák – bobe@chem.u-szeged.hu

*Corresponding author

Experimental procedures for compounds 8–19 and their ^{1}H, ^{13}C NMR, MS, elemental analysis data

Chemistry	S2
General methods for the Sonogashira couplings	S3
Reaction conditions for coupling at C-2	S3
Reaction conditions for coupling at C-4	S8
General method for partial saturation and benzofuran formation	S14
General method for total saturation	S18
Determination of 17β-HSD1 inhibition	S21

Chemistry

Melting points (mp) were determined with a Kofler hot-stage apparatus and are uncorrected. Elemental analyses were performed with a Perkin-Elmer CHN analyzer model 2400. Thin-layer chromatography: silica gel 60 F_{254}; layer thickness 0.2 mm (Merck); in 80% diisopropyl ether/hexane eluent; detection with iodine or UV (365 nm) after spraying with 5% phosphomolybdic acid in 50% aqueous phosphoric acid and heating at 100–120 °C for 10 min. Flash chromatography: silica gel 60, 40–63 µm (Merck). The reactions under microwave irradiation were carried out with a CEM Corporation focused microwave system, Model Discover SP. ^{1}H NMR spectra were recorded in $CDCl_3$ solution (if not otherwise stated) with a Bruker DRX-500 instrument at 500 MHz, with Me_4Si as internal standard. ^{13}C NMR spectra were recorded with the same instrument at 125 MHz under the same conditions. Full scan mass spectra of the compounds were acquired in the range of 50 to 800 *m*/*z* with an Agilent 500MS Ion trap mass spectrometer equipped with an electrospray ionization source. Analyses were performed in positive ion mode. The spectra were collected by continous infusion of the steroid solution at a concentration of 10 ng μl^{-1} in MeCN/5 mM ammonium formate 50/50 (v/v %) at a flow rate of 15 $\mu l\ min^{-1}$. The analytical HPLC measurements were performed on an Agilent 1260 Infinity HPLC equipped with a Micro Vacuum Degasser, Binary Pump, Standard Autosampler, Thermostatted Column Compartment, and Variable Wavelength Detector. The chromatographic separation was achieved on Gemini NX C-18 analytical column (3 µm, 150 × 2mm) from Phenomenex, equipped with a C-18 guard column, at 40 °C using gradient elution. Mobile phase A was water (Sigma Aldrich Ltd. Budapest, Hungary), while mobile phase B was acetonitrile (Merck Ltd. Budapest, Hungary). A linear gradient was applied from 20% B to 100% B in 10 min (holding time: 5 min)

then B content was lowered to 20% in 5 min and finally the column was re-equilibrated for 5 min. The flow rate was set to 0.2 ml/min.

General methods for the Sonogashira couplings

Reaction conditions for coupling at C-2

3-Hydroxy-2-iodo-13α-estra-1,3,5(10)-trien-17-one (**3**, 50 mg, 0.18 mmol) or 2-iodo-3-methoxy-13α-estra-1,3,5(10)-trien-17-one (**4**, 50 mg, 0.12 mmol), $Pd(PPh_3)_4$ (20 mg, 0.018 mmol or 14 mg, 0.012 mmol, 0.1 equiv), CuI (3.4 mg, 0.018 mmol or 2.3 mg, 0.012 mmol, 0.1 equiv) and, respectively THF or DMF (3 ml) were added under nitrogen atmosphere, then Et_3N (0.1 ml, 0.72 mmol, 6 equiv) was added and the mixture was stirred at 50 °C for 10 min. Phenylacetylene (2 equiv) was added with a syringe in 10 mL Pyrex pressure vessels (CEM, Part #: 908035) with silicone cap (CEM, Part #: 909210) and the mixture was heated in a CEM microwave reactor at 50 °C for 20 min under stirring. The solvent was evaporated in vacuo. The residue was purified by flash chromatography with 50% diisopropyl ether/hexane as eluent.

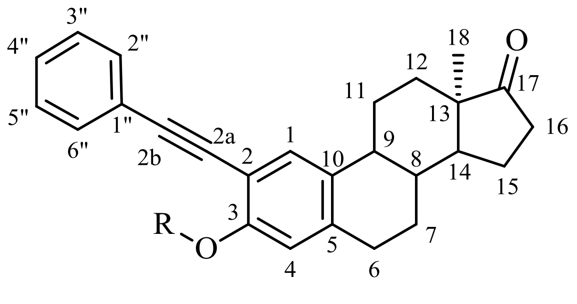

R = H, Me

R_f = 80% diisopropyl ether/hexane

*3-Hydroxy-2-(phenylethynyl)-13α-estra-1,3,5(10)-trien-17-one (**8a**)*

As described in the general method, iodo compound **3** (50 mg, 0.18 mmol) was reacted with phenylacetylene (38 µl, 0.36 mmol). Compound **8a** was obtained as a white solid (38 mg, 86%, Mp.: 159−161°C, R_f = 0.40). Anal. Calcd. for $C_{26}H_{26}O_2$: C,

84.29; H, 7.07. Found: C, 84.35; H, 6.92. ^{1}H NMR (500 MHz, $CDCl_3$) δ ppm : 1.05(s, 3H, 18-H_3); 2.82(m, 2H, 6-H_2); 5.66(s, 1H, OH); 6.68(s, 1H, 4-H); 7.32(s, 1H, 1-H); 7.34 and 7.52(2x overlapping multiplets, 3H and 2H, 2"-H, 3"-H, 4"-H, 5"-H and 6"-H); ^{13}C NMR (500 MHz, $CDCl_3$) δ ppm : 21.01; 25.1(C-18); 28.1; 28.2; 30.3; 31.9; 33.4; 41.2; 41.3; 49.3; 50.1(C-13); 83.5 and 95.4(2x1C, C-2a and C-2b); 107.1(C-2); 114.3(C-4); 122.6(C-1"); 128.4 and 131.5(2x2C: C-2", C-3", C-5", C-6"); 128.5 and 129.1(C-1, C-4"); 132.1(C-10); 140.0(C-5); 154.2(C-3); 221.5(C-17). MS *m/z* (%): 371 (100, $[M+H]^+$).

*3-Hydroxy-2-[(4-methylphenyl)ethynyl]-13α-estra-1,3,5(10)-trien-17-one (**8b**)*

As described in the general method, iodo compound **3** (50 mg, 0.18 mmol) was reacted with 4-methylphenylacetylene (38 µl, 0.36 mmol). Compound **8b** was obtained as a white solid (41 mg, 89%, Mp.: 181−183°C, R_f = 0.45). Anal. Calcd. for $C_{27}H_{28}O_2$: C, 84.34; H, 7.34. Found: C, 84.42; H, 7.26. ^{1}H NMR (500 MHz, DMSO-d_6) δ ppm : 0.96(s, 3H, 18-H_3); 2.32(s, 3H, 4"−CH_3); 2.70(m, 2H, 6-H_2); 6.57(s, 1H, 4-H); 7.20 and 7.38(2xd, 2x2H, *J* = 7.9 Hz, 2"-H, 3"-H, 5"-H, and 6"-H); 7.22(s, 1H, 1-H); 9.59(s, 1H, OH); ^{13}C NMR δ ppm : 20.4; 20.9(4"−CH_3); 24.5(C-18); 27.5; 27.9; 29.6; 31.4; 32.8; 40.4; 40.6; 48.4; 49.3(C-13); 86.6 and 91.6(2x1C; C-2a and C-2b); 107.4(C-2); 114.9(C-4); 120.2(C-1"); 129.1 and 130.9(2x2C: C-2", C-3", C-5", C-6"); 130.1(C-1); 130.4(C-4"); 137.6(C-10); 138.9(C-5); 155.6(C-3); 220.5(C-17). MS *m/z* (%): 385 (100, $[M+H]^+$).

*3-Hydroxy-2-[(4-methoxyphenyl)ethynyl]-13α-estra-1,3,5(10)-trien-17-one (**8c**)*

As described in the general method, iodo compound **3** (50 mg, 0.18 mmol) was reacted with 4-methoxyphenylacetylene (36 µl, 0.36 mmol). Compound **8c** was obtained as a white solid (44 mg, 84%, Mp.: 169−171°C, R_f = 0.29). Anal. Calcd. for $C_{27}H_{28}O_3$: C, 80.97; H, 7.05. Found: C, 81.06; H, 6.83. ^{1}H NMR (500 MHz, DMSO-d_6) δ ppm : 0.96(s, 3H, 18-H_3); 2.70(m, 2H, 6-H_2); 3.77(s, 3H, 4"−OCH_3); 6.57(s, 1H, 4-

H); 6.95 and 7.42(2xd, 2x2H, *J* = 7.8 Hz, 2”-H, 3”-H, 5”-H, 6”-H); 7.20(s, 1H, 1-H); 9.54(s, 1H, OH); ^{13}C NMR δ ppm : 20.4; 24.4(C-18); 27.5; 27.9; 29.5; 31.5; 32.8; 40.4; 40.6; 48.4; 49.3(C-13); 55.1(4”−OCH_3); 85.7 and 91.4(2x1C, C-2a and C-2b); 107.6(C-2); 114.1 and 132.5(2x2C: C-2”, C-3”, C-5”, C-6”); 114.1(C-4); 115.2(C-1”); 129.9(C-1); 130.3(C-10); 138.7(C-5); 155.5(C-3); 158.9(C-4”); 220.5(C-17). MS *m/z* (%): 401 (100, $[M+H]^+$)

*2-[(4-Fluorophenyl)ethynyl]-3-hydroxy-13α-estra-1,3,5(10)-trien-17-one (**8d**)*

As described in the general method, iodo compound **3** (50 mg, 0.18 mmol) was reacted with 4-fluorophenylacetylene (30 µl, 0.36 mmol). Compound **8d** was obtained as a white solid (39 mg, 84%, Mp.: 206−208°C, R_f =0.38). Anal. Calcd. for $C_{26}H_{25}FO_2$: C, 80.39; H, 6.49. Found: C, 80.47; H, 6.32. ^{1}H NMR (500 MHz, DMSO-d_6) δ ppm : 0.96(s, 3H, 18-H_3); 2.71(m, 2H, 6-H_2); 6.58(s, 1H, 4-H); 7.24 and 7.54(2x overlapping multiplets, 3H+2H, 1-H, 2”-H, 3”-H, 5”-H, 6”-H); 9.62(s, 1H, OH); ^{13}C NMR δ ppm : 20.6; 24.5(C-18); 27.5; 27.9; 29.6; 31.5; 32.8; 40.4; 40.5; 48.5; 49.3(C-13); 86.9 and 90.4(2x1C, C-2a and C-2b); 107.1(C-2); 115.0(C-4); 115.7(d, 2C, *J* = 22.0 Hz C-3”, C-5”); 119.6(C-1”); 130.1(C-1); 130.4(C-10); 133.2(d, 2C, *J* = 8.5 Hz C-2”, C-6”); 139.2(C-5); 155.7(C-3); 162.5(d, *J* = 244 Hz; C-4”); 220.4(C-17). MS *m/z* (%): 389 (100, $[M+H]^+$).

*3-Hydroxy-2-{[4-(trifluoromethyl)phenyl]ethynyl}-13α-estra-1,3,5(10)-trien-17-one (**8e**)*

As described in the general method, iodo compound **3** (50 mg, 0.18 mmol) was reacted with 4-(trifluoromethyl)phenylacetylene (58 µl, 0.36 mmol). Compound **8e** was obtained as a white solid (42 mg, 88%, Mp.: 175−177°C, R_f = 0.33). Anal. Calcd. for $C_{27}H_{25}F_3O_2$: C, 73.96; H, 5.75. Found: C, 74.05; H, 5.68. ^{1}H NMR (500 MHz, $CDCl_3$) δ ppm : 1.06(s, 3H, 18-H_3); 2.82(m, 2H, 6-H_2); 5.64(s, 1H, OH); 6.68(s, 1H, 4-H); 7.33(s, 1H, 1-H); 7.61(s, 4H, 2”-H, 3”-H, 5”-H, 6”-H); ^{13}C NMR δ ppm : 21.0; 25.0(C-18); 28.0; 28.2; 30.3; 31.9; 33.4; 41.2; 41.3; 49.3; 50.1(C-13); 86.3 and

93.8(2x1C, C-2a and C-2b); 106.5(C-2); 114.6(C-4); 123.8(d, *J* = 272.2 Hz, CF_3); 125.3 and 131.6(2x2C: C-2”, C-3”, C-5”, C-6”); 126.5(C-1”); 129.4(C-1); 130.1(d, *J* = 32.7 Hz, C-4”); 132.3(C-10); 140.7(C-5); 154.4(C-3); 221.5(C-17). MS *m/z* (%): 439 (100, $[M+H]^+$).

*3-Methoxy-2-(phenylethynyl)-13α-estra-1,3,5(10)-trien-17-one (**9a**)*

As described in the general method, iodo compound **4** (50 mg, 0.12 mmol) was reacted with phenylacetylene (25 µl, 0.24 mmol). Compound **9a** was obtained as a white solid (45 mg, 98%, Mp.: 148−149°C, R_f = 0.46). Anal. Calcd. for $C_{27}H_{28}O_2$: C, 84.34; H, 7.34. Found: C, 84.22; H, 7.38. ^{1}H NMR (500 MHz, DMSO-d_6) δ ppm : 0.97(s, 3H, 18-H_3); 2.81(m, 2H, 6-H_2); 3.80(s, 3H, 3−OCH_3); 6.75(s, 1H, 4-H); 7.33(s, 1H, 1-H); 7.41 and 7.49(2x overlapping multiplets, 3H and 2H, 2”-H, 3”-H, 4”-H, 5”-H, 6”-H); ^{13}C NMR δ ppm : 20.3; 24.4(C-18); 27.4; 27.8; 29.9; 31.5; 32.8; 40.4; 40.5; 48.4; 49.3(C-13); 55.5(3−OCH_3); 86.7 and 91.8(2x1C, C-2a and C-2b); 108.6(C-2); 111.1(C-4); 122.9(C-1”); 128.2(C-4); 128.5 and 131.0(2x2C: C-2”, C-3”, C-5”, C-6”); 130.5(C-1”); 131.6(C-10); 139.5(C-5); 157.3(C-3); 220.4(C-17). MS *m/z* (%): 385 (100, $[M+H]^+$).

*3-Methoxy-2-[(4-methylphenyl)ethynyl]-13α-estra-1,3,5(10)-trien-17-one (**9b**)*

As described in the general method, iodo compound **4** (50 mg, 0.12 mmol) was reacted with 4-methylphenylacetylene (28 µl, 0.24 mmol). Compound **9b** was obtained as a white solid (43 mg, 90%, Mp.: 83−85°C, R_f = 0.48). Anal. Calcd. for $C_{28}H_{30}O_2$: C, 84.38; H, 7.59. Found: C, 84.41; H, 7.65. ^{1}H NMR (500 MHz, DMSO-d_6) δ ppm : 0.96(s, 3H, 18-H_3); 2.32(s, 3H, 4”−CH_3); 2.80(m, 2H, 6-H_2); 3.79(s, 3H, 3−OCH_3); 6.73(s, 1H, 4-H); 7.20 and 7.37(2xd, 2x2H, *J* = 7.9 Hz, 2”-H, 3”-H, 5”-H, 6”-H); 7.31(s, 1H, 1-H); ^{13}C NMR δ ppm : 20.3; 20.9(4”−CH_3); 24.4(C-18); 27.4; 27.9; 29.9; 31.5; 32.8; 40.4; 40.5; 48.4; 49.3(C-13); 55.4(3−OCH_3); 86.0 and 91.9(2x1C C-2a and C-2b); 108.8(C-2); 111.0(C-4); 119.9(C-1”); 129.1 and 130.9(2x2C: C-3”, C-

5”, C-2”, C-6”); 130.4(C-1); 131.5(C-10); 137.8(C-4”); 139.2(C-5); 157.1(C-3); 220.4(C-17). MS *m/z* (%): 105 (66), 399 (100, $[M+H]^+$).

*3-Methoxy-2-[(4-methoxyphenyl)ethynyl]-13α-estra-1,3,5(10)-trien-17-one (**9c**)*

As described in the general method, iodo compound **4** (50 mg, 0.12 mmol) was reacted with 4-methoxyphenylacetylene (31 µl, 0.24 mmol). Compound **9c** was obtained as a white solid (41 mg, 83%, Mp.: 77−79°C, R_f = 0.38). Anal. Calcd. for $C_{28}H_{30}O_3$: C, 81.13; H, 7.29. Found: C, 81.25; H, 7.21. ^{1}H NMR (500 MHz, DMSO-d_6) δ ppm : 0.97(s, 3H, 18-H_3); 2.80(m, 2H, 6-H_2); 3.78(s, 6H, 3−OCH_3 and 4”−OCH_3); 6.73(s, 1H, 4-H); 6.95 and 7.42(2xd, 2x2H, *J* = 8.7 Hz, 2”-H, 3”-H, 5”-H, 6”-H); 7.30(s, 1H, 1-H); ^{13}C NMR δ ppm : 20.3; 24.4(C-18); 27.4; 27.9; 29.9; 31.5; 32.8; 40.4; 40.5; 48.4; 49.3(C-13); 55.1 and 55.4(2C: 3−OCH_3 and 4”−OCH_3); 85.1 and 91.8(2x1C C-2a and C-2b); 109.0(C-2); 111.0(C-4); 114.2 and 132.5(2x2C: C-2”, C-3”, C-5”, C-6”); 114.9(C-1”); 130.3(C-1); 131.5(C-10); 132.5(2C: C-2”, C-6”); 139.0(C-5); 157.1 and 159.1(C-3 and C-4”); 220.4(C-17). MS *m/z* (%):400 (100), 415 (80, $[M+H]^+$).

*2-[(4-Fluorophenyl)ethynyl]-3-methoxy-13α-estra-1,3,5(10)-trien-17-one (**9d**)*

As described in the general method, iodo compound **4** (50 mg, 0.12 mmol) was reacted with 4-fluorophenylacetylene (27 µl, 0.24 mmol). Compound **9d** was obtained as a white solid (47 mg, 97%, Mp.: 162−164 °C, R_f = 0.44). Anal. Calcd. for $C_{27}H_{27}FO_2$: C, 80.57; H, 6.76. Found: C, 80.47; H, 6.82. ^{1}H NMR (500MHz, DMSO-d_6) δ ppm : 0.96(s, 3H, 18-H_3); 2.81(m, 2H, 6-H_2); 3.79(s, 3H, 3−OCH_3); 6.74(s, 1H, 4-H); 7.24 and 7.54(2xdd, 2x2H, *J* = 8.7 Hz, *J* = 5.7 Hz, 2”-H, 3”-H, 5”-H, 6”-H); 7.33(s, 1H, 1-H); ^{13}C NMR δ ppm: 20.5; 24.6(C-18); 27.6; 28.0; 30.1; 31.7; 32.9; 40.5; 40.6; 48.6; 49.5(C-13); 55.6(3−OCH_3); 86.6 and 90.9(2x1C C-2a and C-2b); 108.6(C-2); 111.2(C-4); 115.9(2C, d, *J* = 22 Hz, C-3” and C-5”); 119.5(C-1”); 130.6(C-1); 131.8(C-10); 133.4(2C, d, *J* = 8.4 Hz, C-2”, and C-6”); 139.7(C-5); 157.4(C-3); 161.8(d, *J* = 247 Hz, C-4”); 220.6(C-17). MS *m/z* (%): 403 (100, $[M+H]^+$).

*3-Methoxy-2-{[4-(trifluoromethyl)phenyl]ethynyl}-13α-estra-1,3,5(10)-trien-17-one (**9e**)*

As described in the general method, iodo compound **4** (50 mg, 0.12 mmol) was reacted with 4-(trifluoromethyl)phenylacetylene (39 µl, 0.24 mmol). Compound **9e** was obtained as a white solid (44 mg, 81%, Mp.: 131−132°C, R_f = 0.42). Anal. Calcd. for $C_{28}H_{27}F_3O_2$: C, 74.32; H, 6.01 Found: C, 74.19; H, 5.93. ^{1}H NMR (500 MHz, DMSO-d_6) δ ppm : 0.97(s, 3H, 18-H_3); 2.82(m, 2H, 6-H_2); 3.81(s, 3H, 3−OCH_3); 6.78(s, 1H, 4-H); 7.39(s, 1H, 1-H); 7.69(d; *J* = 8.1Hz; 2H, 2"-H, 6"-H); 7.75(d; *J* = 8.1 Hz, 2H, 3"-H, 5"-H); ^{13}C NMR δ ppm : 20.4; 24.5(C-18); 27.4; 27.9; 30.0; 31.5; 32.8; 40.4; 48.4; 49.4(C-13); 55.6(3−OCH_3); 89.6(2a); 90.5(2b); 107.9(C-2); 111.2(C-4); 123.8(d, *J* = 272.2 Hz, CF_3) 125.5 and 131.7(2x2C: C-2", C-3", C-5", C-6"); 126.5(C-1"); 130.1(d, *J* = 32.8 Hz, C-4") 130.8(C-1); 131.8(C-10); 140.3(C-5); 157.5(C-3); 220.5(C-17). MS *m/z* (%):289 (44), 453 (100, $[M+H]^+$).

Reaction conditions for coupling at C-4

3-Hydroxy-4-iodo-13α-estra-1,3,5(10)-trien-17-one (**5**, 50 mg, 0.18 mmol) or 4-iodo-3-methoxy-13α-estra-1,3,5(10)-trien-17-one (**6**, 50 mg, 0.12 mmol), $Pd(PPh_3)_2Cl_2$ (6.3 mg, 0.009 mmol, or 4.2 mg, 0.006 mmol, 0.05 equiv), CuI (1.7 mg, 0.009 mmol, or 1.1 mg, 0.006 mmol, 0.05 equiv), Et_3N (0.1 ml, 0.72 mmol, 6 equiv) and respectively CH_3CN or DMF (3 ml) were added under nitrogen atmosphere and stirred for 2 min in 10 mL Pyrex pressure vessels (CEM, Part #: 908035) with silicone cap (CEM, Part #: 909210). Phenylacetylene (2 equiv) was added with a syringe and the mixture was heated in a CEM microwave reactor at 80 °C for 20 min under stirring. The solvent was evaporated in vacuo. The residue was purified by flash chromatography with 30% diisopropyl ether/hexane as eluent.

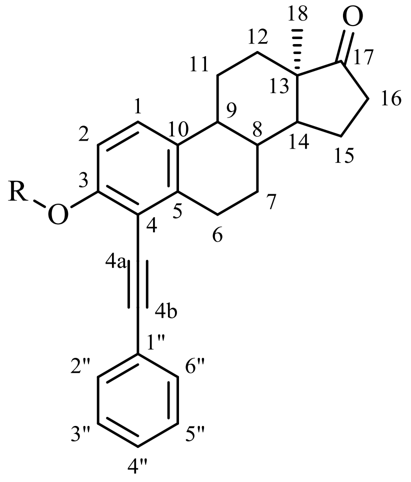

R = H, Me

*3-Hydroxy-4-(phenylethynyl)-13α-estra-1,3,5(10)-trien-17-one (**10a**)*

As described in the general method, iodo compound **5** (50 mg, 0.18 mmol) was reacted with phenylacetylene (38 µl, 0.36 mmol). Compound **10a** was obtained as a white solid (36 mg, 81%, Mp.: 184−186°C, R_f = 0.53). Anal. Calcd. for $C_{26}H_{26}O_2$: C, 84.29; H, 7.07. Found: C, 84.35; H, 6.98. ^{1}H NMR (500 MHz, DMSO-d_6) δ ppm : 0.96(s, 3H, 18-H_3); 2.76 and 3.02(2xm, 2x1H, 6-H_2); 6.69(d, 1H, *J* = 8.5 Hz, 2-H); 7.12(d, 1H, *J* = 8.5 Hz, 1-H); 7.39 and 7.50(2x overlapping multiplets, 3H and 2H, 2”-H, 3”-H, 4”-H, 5”-H, 6”-H); 9.62(s, 1H OH); ^{13}C NMR δ ppm : 20.4; 24.4(C-18); 27.5; 27.9; 28.8; 31.5; 32.8; 40.2; 40.7; 48.3; 49.3(C-13); 85.4 and 97.3(2x1C, C-4a and C-4b); 108.7(C-4); 112.7(C-2); 123.3(C-1”); 127.1 and 128.0(C-1, C-4”); 128.5 and 130.9(2x2C: C-2”, C-3”, C-5” C-6”); 130.4(C-10); 139.5(C-5); 156.2(C-3); 220.6(C-17). MS *m/z* (%):

*3-Hydroxy-4-[(4-methylphenyl)ethynyl]-13α-estra-1,3,5(10)-trien-17-one (**10b**)*

As described in the general method, iodo compound **5** (50 mg, 0.18 mmol) was reacted with 4-methylphenylacetylene (38 µl, 0.36 mmol). Compound **10b** was obtained as a white solid (37 mg, 80%, Mp.: 172−174°C, R_f = 0.55). Anal. Calcd. for $C_{27}H_{28}O_2$: C, 84.34; H, 7.34. Found: C, 84.26; H, 7.22. ^{1}H NMR (500 MHz, DMSO-d_6)

δ ppm : 0.96(s, 3H, 18-H_3); 2.33(s, 3H, 4”−OCH_3); 2.74 and 3.01(2xm, 2x1H, 6-H_2); 6.68(d, 1H, *J* = 8.5 Hz, 2-H); 7.11(d, 1H, *J* = 8.5 Hz, 1-H); 7.21 and 7.38(2xd, 2x2H, *J* = 7.7 Hz, 2”-H, 3”-H, 5”-H, 6”-H); 9.57(s, 1H, OH); ^{13}C NMR δ ppm : 20.4; 20.9(4”−CH_3); 24.4(C-18); 27.5; 27.9; 28.8; 31.5; 32.8; 40.2; 40.7; 48.3; 49.3(C-13); 84.7 and 97.4(2x1C, C-4a and C-4b); 108.9(C-4); 112.6(C-2); 120.3(C-4”); 126.9(C-1); 129.1 and 130.8 (2x2C: C-2”, C-3”, C-5”, C-6”); 130.3(C-10); 137.7(C-1”); 139.4(C-5); 156.1(C-3); 220.6(C-17). MS *m/z* (%): 385 (100, $[M+H]^+$).

*3-Hydroxy-4-[(4-methoxyphenyl)ethynyl]-13α-estra-1,3,5(10)-trien-17-one (**10c**)*

As described in the general method, iodo compound **5** (50 mg, 0.18 mmol) was reacted with 4-methoxyphenylacetylene (36 µl, 0.36 mmol). Compound **10c** was obtained as a white solid (41 mg, 85%, Mp.: 176−178°C, R_f = 0.39). Anal. Calcd. for $C_{27}H_{28}O_3$: C, 80.97; H, 7.05. Found: C, 81.10; H, 7.15. ^{1}H NMR (500 MHz, $CDCl_3$) δ ppm : 1.05(s, 3H, 18-H_3); 2.82 and 3.12(2xm, 2x1H, 6-H_2); 3.83(s, 3H, 4”−OCH_3); 5.77(s, 1H, OH); 6.78(d, 1H, *J* = 8.5 Hz, 2-H); 6.88 and 7.45(2xd, 2x2H, *J* = 7.1 Hz, 2”-H, 3”-H, 5”-H, 6”-H); 7.17(d, 1H, *J* = 8.5 Hz, 1-H); ^{13}C NMR δ ppm : 21.0; 25.1(C-18); 28.1; 28.3; 29.3; 32.0; 33.4; 41.1; 41.5; 49.2; 50.1(C-13); 55.3(4”−OCH_3); 80.7 and 100.7(2x1C, C-4a and C-4b); 109.1(C-4); 111.7(C-2); 114.1 and 132.9(2x2C: C-2”, C-3”, C-5”, C-6”); 114.7(C-4”); 127.3(C-1); 131.7(C-10); 138.9(C-5); 154.5(C-3); 159.9(C-4”); 221.6(C-17). MS *m/z* (%): 401 (100, $[M+H]^+$).

*4-[(4-Fluorophenyl)ethynyl]-3-hydroxy-13α-estra-1,3,5(10)-trien-17-one (**10d**)*

As described in the general method, iodo compound **5** (50 mg, 0.18 mmol) was reacted with 4-fluorophenylacetylene (30 µl, 0.36 mmol). Compound **10d** was obtained as a white solid (37 mg, 79%, Mp.: 175−177°C, R_f = 0.35). Anal. Calcd. for $C_{26}H_{25}FO_2$: C, 80.39; H, 6.49. Found: C, 80.49; H, 6.57. ^{1}H NMR (500 MHz, DMSO-d_6) δ ppm : 0.96(s, 3H, 18-H_3); 2.75 and 3.01(2xm, 2x1H, 6-H_2); 6.68(d, 1H, *J* = 8.5 Hz, 2-H); 7.12(d, 1H, *J* = 8.5 Hz, 1-H); 7.24 and 7.54(2x multiplets, 2x2H, 2”-H, 3”-H,

5”-H, 6”-H); 9.61(s, 1H, OH); ^{13}C NMR δ ppm : 20.4; 24.4(C-18); 27.5; 27.9; 28.8; 31.5; 32.8; 40.2; 40.7; 48.3; 49.3(C-13); 85.1 and 96.1(2x1C, C-4a and C-4b); 108.6(C-4); 112.7(C-2); 115.8(d, 2C, *J* = 22.1 Hz C-3” and C-5”); 119.7(C-1”); 127.2(C-1); 130.3(C-10); 133.1(d, 2C, *J* = 8.5 Hz, C-2” and C-6”); 139.5(C-5); 156.3(C-3); 161.6(d, *J* = 247 Hz, C-4”); 220.6(C-17). MS *m/z* (%): 123 (53), 389(100, $[M+H]^+$).

*3-Hydroxy-4-{[4-(trifluoromethyl)phenyl]ethynyl}-13α-estra-1,3,5(10)-trien-17-one (**10e**)*

As described in the general method, iodo compound **5** (50 mg, 0.18 mmol) was reacted with 4-(trifluoromethyl)phenylacetylene (58 µl, 0.36 mmol). Compound **10e** was obtained as a white solid (43 mg, 82%, Mp.: 199−201°C, R_f = 0.41). Anal. Calcd. for $C_{27}H_{25}F_3O_2$: C, 73.96; H, 5.75. Found: C, 73.87; H, 5.63. ^1H NMR (500 MHz, DMSO-d_6) δ ppm : 0.96(s, 3H, 18-H_3); 2.77 and 3.03(2xm, 2x1H, 6-H_2); 6.72(d, 1H, *J* = 8.5 Hz, 4-H); 7.16(d, 1H, *J* = 8.5 Hz, 1-H); 7.70(d, 2H, *J* = 7.9 Hz, 2”-H, 6”-H); 7.76(d, 2H, *J* = 7.9 Hz, 3”-H, 5”-H); 9.75(s, 1H, OH); ^{13}C NMR δ ppm : 20.4; 24.4(C-18); 27.5; 27.9; 28.8; 31.5; 32.8; 40.1; 40.7; 48.3; 49.3(C-13); 88.3 and 95.8(2x1C, C-4a and C-4b); 108.2(C-4); 112.8(C-2); 125.4 and 125.5 and 131.6(1C+1C+2C, C-2”, C-3”, C-5”, C-6”); 127.5(C-1”); 123.8(d, *J* = 272.2 Hz, CF_3), 127.8(C-1); 130.4(C-10); 139.5(C-5); 133.7(d, *J* = 32.8 Hz, C-4”); 156.6(C-3); 220.5(C-17). MS *m/z* (%): 439 (100, $[M+H]^+$),

*3-Methoxy-4-(phenylethynyl)-13α-estra-1,3,5(10)-trien-17-one (**11a**)*

As described in the general method, iodo compound **6** (50 mg, 0.12 mmol) was reacted with phenylacetylene (25 µl, 0.24 mmol). Compound **11a** was obtained as a white solid (42 mg, 91%, Mp.: 78−80°C, R_f = 0.63). Anal. Calcd. for $C_{27}H_{28}O_2$: C, 84.34; H, 7.34. Found: C, 84.48; H, 7.50. ^1H NMR (500 MHz, DMSO-d_6) δ ppm : 0.96(s, 3H, 18-H_3); 2.78 and 3.04(2xm, 2x1H, 6-H_2); 3.79(s, 3H, 3−OCH_3); 6.86(d,

1H, *J* = 8.8 Hz, 2-H); 7.30(d, 1H, *J* = 8.8 Hz, 1-H), 7.40(2x overlapping multiplets, 3H and 2H, 2”-H, 3”-H, 4”-H, 5”-H, 6”-H); ^{13}C NMR δ ppm : 20.4; 24.4(C-18); 27.4; 27.9; 28.7; 31.5; 32.8; 40.1; 40.7; 48.3; 49.3(C-13); 55.5(3−OCH_3); 84.8 and 97.8(2x1C, C-4a and C-4b); 108.4(C-2); 110.1(C-4); 123.0(C-1”); 127.2 and 128.2(C-1 and C-4”); 128.5 and 130.9(2x2C: C-2”, C-3”, C-5”, C-6”); 132.0(C-10); 140.0(C-5); 157.7(C-3); 220.5(C-17). MS *m/z* (%): 385 (100, $[M+H]^+$).

*3-Methoxy-4-[(4-methylphenyl)ethynyl]-13α-estra-1,3,5(10)-trien-17-one (**11b**)*

As described in the general method, iodo compound **6** (50 mg, 0.12 mmol) was reacted with 4-methylphenylacetylene (28 µl, 0.24 mmol). Compound **11b** was obtained as a white solid (36 mg, 75%, Mp.: 121−123°C, R_f = 0.69). Anal. Calcd. for $C_{28}H_{30}O_2$: C, 84.38; H, 7.59. Found: C, 84.52; H, 7.38. ^{1}H NMR (500 MHz, DMSO-d_6) δ ppm : 0.96(s, 3H, 18-H_3); 2.32(s, 3H, 4”−CH_3); 2.76 and 3.02(m, 2H, 6-H_2); 3.78(s, 3H, 3−OCH_3); 6.83(d, 1H, *J* = 8.8 Hz, 2-H); 7.21 and 7.37(2xd, 2x2H, *J* = 7.9 Hz, 2”-H, 3”-H, 5”-H, 6”-H); 7.26(d, 1H, *J* = 8.8 Hz, 1-H); ^{13}C NMR δ ppm : 20.6; 21.1; 24.4(C-18); 27.6; 28.1; 29.0; 31.6; 33.0; 40.3; 40.9; 48.5; 49.4(C-13); 55.7(OCH_3); 84.3 and 98.2(2x1C, C-4a and C-4b); 108.6(C-2); 110.5(C-4); 120.2(C-1”); 127.2(C-1); 129.4 and 131.0(2x2C: C-2”, C-3”, C-5”, C-6”); 132.2(C-10); 138.1(C-5); 140.1(C-4”); 157.9(C-3); 220.7(C-17). MS *m/z* (%): 399 (100, $[M+H]^+$).

*3-Methoxy-4-[(4-methoxyphenyl)ethynyl]-13α-estra-1,3,5(10)-trien-17-one (**11c**)*

As described in the general method, iodo compound **6** (50 mg, 0.12 mmol) was reacted with 4-methoxyphenylacetylene (31 µl, 0.24 mmol). Compound **11c** was obtained as a white solid (44 mg, 89%, Mp.: 138−140 °C, R_f = 0.55). Anal. Calcd. for $C_{28}H_{30}O_3$: C, 81.13; H, 7.29. Found: C, 80.92; H, 7.40. ^{1}H NMR (500 MHz, DMSO-d_6) δ ppm : 0.97(s, 3H, 18-H_3); 2.76 and 3.02(2xm, 2x1H, 6-H_2); 3.79(s, 6H, 3−OCH_3 and 4”−OCH_3); 6.83(d, 1H, *J* = 8.7 Hz, 2-H); 6.98 and 7.42(2xd, 2x2H, *J* = 8.8 Hz, 2”-H, 3”-H, 5”-H, 6”-H); 7.26(d, 1H, *J* = 8.7 Hz, 1-H); ^{13}C NMR δ ppm : 20.4; 24.4(C-18);

27.5; 28.0; 28.8; 31.5; 32.9; 40.1; 40.8; 48.3; 49.3(C-13); 55.2 and 55.5(3−OCH_3 and 4”−OCH_3); 83.4 and 97.9(2x1C, C-4a and C-4b); 108.4(C-2); 110.6(C-4); 114.3 and 132.5(2x2C: C-2”, C-3”, C-5”, C-6”); 115.0(C-1”); 126.9(C-1); 132.0(C-10); 139.8(C-5); 157.6 and 159.1(C-3 and C-4”); 220.6(C-17). MS *m/z* (%): 415 (100, $[M+H]^+$).

*4-[(4-Fluorophenyl)ethynyl]-3-methoxy-13α-estra-1,3,5(10)-trien-17-one (**11d**)*

As described in the general method, iodo compound **6** (50 mg, 0.12 mmol) was reacted with 4-fluorophenylacetylene (27 µl, 0.24 mmol). Compound **11d** was obtained as a white solid (38 mg, 79%, Mp.: 110−112°C, R_f =0.66). Anal. Calcd. for $C_{27}H_{27}FO_2$: C, 80.57; H, 6.76. Found: C, 80.67; H, 6.59. ^{1}H NMR (500 MHz, DMSO-d_6) δ ppm : 0.97(s, 3H, 18-H_3); 2.77 and 3.03(2xm, 2x1H, 6-H_2); 3.79(s, 3H, 3−OCH_3); 6.85(d, 1H, *J* = 8.7 Hz, 2-H); 7.24(d, 1H, 1-H); 7.26(dd, 2H, *J* = 13.6 Hz, *J* = 8.8 Hz, 3”-H, 5”-H); 7.54(dd, 2H, *J* = 8.8 Hz, *J* = 5.5 Hz, 2”-H, 6”-H) ^{13}C NMR δ ppm : 20.4; 24.4(C-18); 27.4; 27.9; 28.8; 31.5; 32.8; 40.1; 40.7; 48.3; 49.3(C-13); 55.5(3−OCH_3); 84.5 and 96.7(2x1C, C-4a and C-4b); 108.4(C-2); 110.0(C-4); 115.8(d, 2C, *J* = 22.1 Hz, C-3”, C-5”); 119.4(C-1); 127.3(C-1”); 132.0(C-10); 133.2(d, 2C, *J* = 8.5 Hz, C-2”, C-6”); 140.0(C-5); 157.8(C-3); 161.7(d, *J* = 247 Hz, C-4”); 220.5(C-17). MS *m/z* (%): 403 (100, $[M+H]^+$).

*3-Methoxy-4-{[4-(trifluoromethyl)phenyl]ethynyl}-13α-estra-1,3,5(10)-trien-17-one (**11e**)*

As described in the general method, iodo compound **6** (50 mg, 0.12 mmol) was reacted with 4-(trifluoromethyl)phenylacetylene (39 µl, 0.24 mmol). Compound **11e** was obtained as a white solid (38 mg, 76%, Mp.: 153−155°C, R_f = 0.66). Anal. Calcd. for $C_{28}H_{27}F_3O_2$: C, 74.32; H, 6.01. Found: C, 74.21; H, 5.93. ^{1}H NMR (500 MHz, DMSO-d_6) δ ppm : 0.97(s, 3H, 18-H_3); 2.81 and 3.04(2xm, 2x1H, 6-H_2); 3.81(s, 3H, 3−OCH_3); 6.88(d, 1H, *J* = 8.7 Hz, 2-H); 7.33(d, 1H, *J* = 8.7 Hz, 1-H); 7.70 and 7.77(2x overlapping multiplets, 2x2H, 2”-H, 3”-H, 5”-H, 6”-H); ^{13}C NMR δ ppm : 20.4;

24.4(C-18); 27.4; 27.9; 28.8; 31.5; 32.9; 40.1; 40.7; 48.3; 49.3(C-13); 55.6(OCH_3); 87.7 and 96.4(2x1C, C-4a and C-4b); 108.5(C-2); 109.4(C-4); 120.1(C-1”); 125.5 and 131.7(2x2C: C-2”, C-3”, C-5”, C-6”); 127.3(C-1); 128.0(C-4”); 132.2(C-10); 140.4(C-5); 158.1(C-3); 220.6(C-17). MS *m/z* (%): 453 (100, $[M+H]^+$).

General method for partial saturation and benzofuran formation

3-Hydroxy-2- or 3-hydroxy-4-[(4-methoxyphenyl)ethynyl]-13α-estra-1,3,5(10)-trien-17-one (**8c** or **10c**, 48 mg, 0.12 mmol) or 3-methoxy-2- or 3-methoxy-4-[(4-methoxyphenyl)ethynyl]-13α-estra-1,3,5(10)-trien-17-one (**9c** or **11c**, 50 mg, 0.12 mmol) was dissolved in dry DMF (3 ml), $Pd(OAc)_2$ or $Pd(PPh_3)_2Cl_2$ (2.7 mg, 0.012 mmol or 2.8 mg, 0.004 mmol) as catalyst and KOH (112.0 mg, 2.0 mmol) was added under N_2 atmosphere in 10 mL Pyrex pressure vessels (CEM, Part #: 908035) with silicone cap (CEM, Part #: 909210). The mixture was stirred in a CEM microwave reactor at 50 W for 35 min. The solvent was removed under reduced pressure and the crude product was subjected to flash chromatography with 30% diisopropyl ether/hexane as eluent.

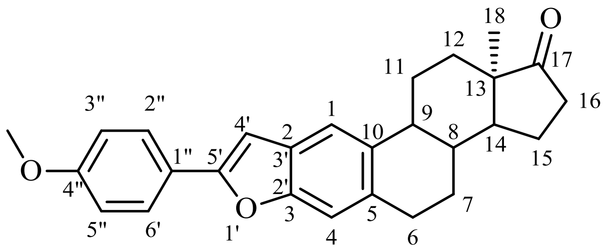

*5’-(4”-Methoxyphenyl)furo[3’,2’:2,3]-13α-estra-1,3,5(10)-trien-17-one (**12**)*

As described in the general method, compound **8c** (50 mg, 0.18 mmol) was reacted under microwave conditions. Compound **12** was obtained as a white solid (44 mg, 92%, 95−97°C, R_f = 0.58). Anal. Calcd. for $C_{27}H_{28}O_3$: C, 80.97; H, 7.05. Found: C,

80.81; H, 7.21. ^{1}H NMR (500 MHz, $CDCl_3$) δ ppm : 1.07(s, 3H, 18-H_3); 2.96(m, 2H, 6-H_2); 3.85(s, 3H, 4”−OCH_3); 6.79(s, 1H, 4’−H); 6.96(d, 2H, J = 8.3 Hz, 2”-H, 6”-H); 7.19(s, 1H, 4-H); 7.43(s, 1H, 1-H); 7.76(d, 2H, J = 8.3 Hz, 3”-H, 5”-H); ^{13}C NMR δ ppm : 21.2; 25.1(C-18); 28.4; 28.7; 30.6; 32.1; 33.5; 41.4; 41.8; 49.7; 50.2(C-13); 55.3(4”−OCH_3), 99.6(C-4’); 110.1(C-4); 114.2 and 126.2(2x2C: C-2”, C-3”, C-5”, C-6”); 117.2(C-1); 123.6(C-2); 127.6(C-1”); 133.6(C-5); 134.9(C-10); 153.4 and 155.6(C-3 and C-5’); 159.8(C-4”); 221.7(C-17). MS *m/z* (%): 304 (53), 332 (52), 401 (100, $[M+H]^+$).

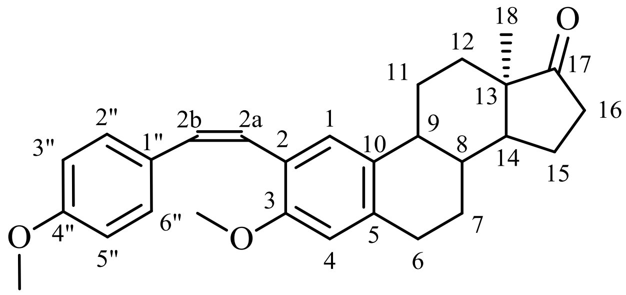

(Z)-3-Methoxy-2-[(4-methoxyphenyl)ethenyl]-13α-estra-1,3,5(10)-trien-17-one (***13***)

As described in the general method, compound **9c** (50 mg, 0.12 mmol) was reacted with DMF and KOH as hydrogen source under microwave conditions. Compound **13** was obtained as an oil (42 mg, 84%, R_f = 0.55). Anal. Calcd. for $C_{28}H_{32}O_3$: C, 80.73; H, 7.74. Found: C, 80.92; H, 7.59. ^{1}H NMR (500 MHz, $CDCl_3$) δ ppm : 1.00(s, 3H, 18-H_3); 2.82(m, 2H, 6-H_2); 3.77 and 3.79(2xs, 2x3H, 3−OCH_3 and 4”−OCH_3); 6.52(s, 2H, 2a-H and 2b-H); 6.56(s, 1H, 4-H); 6.71 and 7.18(2xd, 2x2H, J = 8.6 Hz, 2”-H, 3”-H, 5”-H, 6”-H); 7.15(s, 1H, 1-H); ^{13}C NMR δ ppm : 20.5; 24.7(C-18); 27.7; 27.8; 30.1; 31.6; 33.0; 40.9; 41.2; 48.8; 49.7(C-13); 54.8 and 55.1(4”−OCH_3 and 3−OCH_3); 110.3(C-4); 113.1 and 129.5(2x2C: C-2”, C-3”, C-5”, C-6”); 121.5(C-2); 123.6(C-1); 127.4 and 128.8(2x1C, C-2a and C-2b); 129.6(C-1”); 130.8(C-10); 136.6(C-5); 154.7 and 158.1(C-3 and C-4”); 221.1(C-17). MS *m/z* (%): 417 (100, $[M+H]^+$).

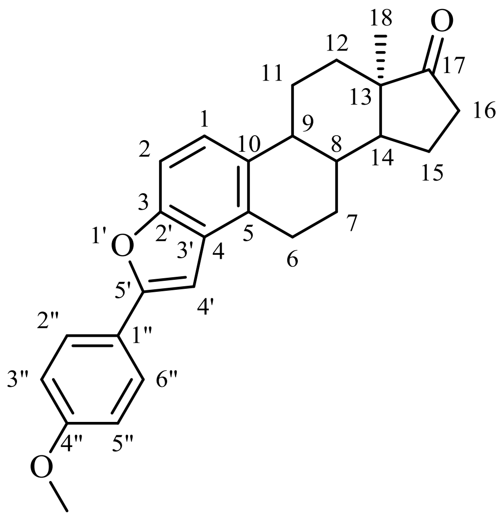

*5'-(4"-Methoxyphenyl)furo[2',3':3,4]-13α-estra-1,3,5(10)-trien-17-one (**14**)*

As described in the general method, compound **10c** (50 mg, 0.12 mmol) was reacted under microwave conditions. Compound **14** was obtained as a white solid (45 mg, 94%, 147−150°C, R_f = 0.51). Anal. Calcd. for $C_{27}H_{28}O_3$: C, 80.97; H, 7.05. Found: C, 80.82; H, 6.97. ^{1}H NMR (500 MHz, $CDCl_3$) δ ppm : 1.08(s, 3H, 18-H_3); 2.95 and 3.08(2xm, 2x1H, 6-H_2); 3.86(s, 3H, 4"−OCH_3); 6.83(s, 1H, 4'−H); 6.96 and 7.78(2xd, 2x2H, *J* = 8.7 Hz, 2"-H, 3"-H, 5"-H, 6"-H); 7.19(d, 1H, *J* = 8.6 Hz, 1-H); 7.28(d, 1H, *J* = 8.6 Hz, 2-H); ^{13}C NMR δ ppm : 21.1; 25.1(C-18); 27.3; 27.9; 28.8; 32.3; 33.5; 41.6; 42.0; 49.3; 50.1(C-13); 55.3(4"−OCH_3); 98.1(C-4'); 108.4(C-2); 114.2 and 126.2(2x2C: C-2", C-3", C-5", C-6"); 121.9(C-1); 123.5(C-4); 128.5(C-1"); 128.9(C-5); 133.5(C-10); 152.7 and 155.8(C-5' and C-3); 159.8(C-4"); 221.6(C-17). MS *m/z* (%):304 (73), 332 (72), 401 (100, $[M+H]^+$).

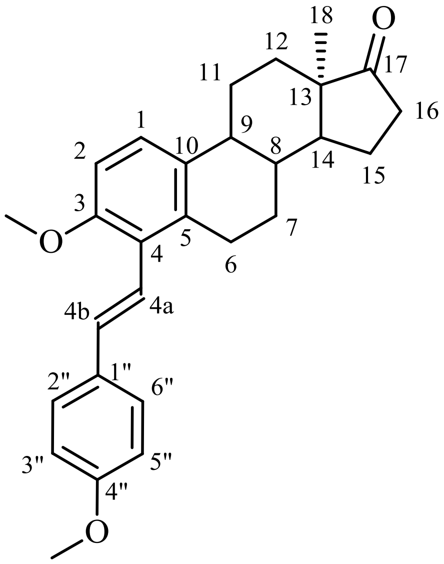

*(E)-3-Methoxy-4-[(4-methoxyphenyl)ethenyl]-13α-estra-1,3,5(10)-trien-17-one (**15**)*

As described in the general method, compound **11c** (50 mg, 0.12 mmol) was reacted with DMF and KOH as hydrogen source under microwave conditions. Compound **15** was obtained as an oil (38 mg, 76%, R_f = 0.60). Anal. Calcd. for $C_{28}H_{32}O_3$: C, 80.73; H, 7.74. Found: C, 80.89; H, 7.65. ^{1}H NMR (500 MHz, $CDCl_3$) δ ppm : 1.04(s, 3H, 18-H_3); 2.47 and 2.73(2xm, 2x1H, 6-H_2); 3.66 and 3.74(2xs, 2x3H, 3−OCH_3 and 4”−OCH_3); 6.30 and 6.61(2xd, 2x1H, *J* = 12.2 Hz, 2a-H and 2b-H); 6.67 and 6.97(2xd, 2x2H, *J*= 8.6 Hz, 2”-H, 3”-H, 5”-H, 6”-H); 6.73(d, 1H, *J* = 8.6 Hz, 2-H); 7.21(d, 1H, *J* = 8.6 Hz, 1-H); ^{13}C NMR δ ppm : 20.9; 25.0(C-18); 27.7; 28.3; 28.4; 29.7; 32.1; 33.4; 40.9; 41.6; 49.5; 50.1(C-13); 55.1 and 55.5(4”−OCH_3 and 3−OCH_3); 108.4(C-2); 113.4 and 129.3(2x2C: C-2”, C-3”, C-5”, C-6”); 123.0(C-1); 125.3 and 130.9(2x1C, C-2a and C-2b); 125.6(C-4); 130.5(C-10); 132.6(C-1”); 136.2(C-5); 154.7(C-3); 158.6(C-4”); 221.7(C-17). MS *m/z* (%): 417 (100, $[M+H]^+$).

General method for total saturation

Similar as already described in [2] a suspension of 3-hydroxy-2- or 3-hydroxy-4-[(4-methoxyphenyl)ethynyl]-13α-estra-1,3,5(10)-trien-17-one (**8c** or **10c**, 48 mg, 0.12 mmol) or 3-methoxy-2- or 3-methoxy-4-[(4-methoxyphenyl)ethynyl]-13α-estra-1,3,5(10)-trien-17-one (**9c** or **11c**, 50 mg, 0.12 mmol) and Pd/C (72 mg, 10%) in ethyl acetate (30 ml). was subjected to 20 bar of H_2 pressure at room temperature for 1 h. The catalyst was then removed by filtration through a short pad of Celite®. After evaporation of the solvent in vacuo, the crude product was subjected to flash chromatography with 30% diisopropyl ether/hexane as eluent..

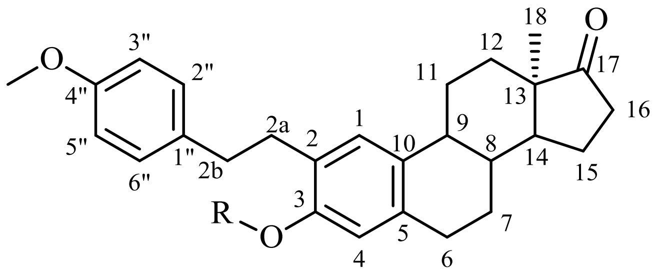

R = H, Me

*3-Hydroxy-2-[(4-methoxyphenyl)ethyl]-13α-estra-1,3,5(10)-trien-17-one (**16**)*

As described in the general method, compound **8c** (50 mg, 0.12 mmol) was reacted with elemental hydrogen under catalytic conditions in an autoclave. Compound **16** was obtained as a white solid (39 mg, 80%, Mp.: 134−136°C, R_f = 0.42). Anal. Calcd. for $C_{27}H_{32}O_3$: C, 80.16; H, 7.97. Found: C, 80.29; H, 8.06. ^{1}H NMR (500 MHz, $CDCl_3$) δ ppm : 1.06(s, 3H, 18-H_3); 2.74(m, 2H, 6-H_2); 2.81(m, 4H, 2a-H_2 and 2b-H_2); 3.79(s, 3H, 4”−OCH_3); 4.82(s, 1H, OH); 6.47(s, 1H, 4-H); 6.83(d, 2H, *J* = 7.2 Hz, 3”-H, 5”-H); 6.97(s, 1H 1-H); 7.12(d, 2H, *J* = 7.2 Hz, 2”-H, 6”-H); ^{13}C NMR δ ppm : 21.0; 25.1(C-18); 28.3; 28.4; 29.8; 32.1; 32.6; 33.5; 35.6; 41.4; 41.6; 49.2; 50.2(C-13); 55.3(4”−OCH_3); 113.8 and 129.3(2x2C: C-2”, C-3”, C-5”, C-6”); 115.1(C-4); 125.5(C-

2); 127.7(C-1); 131.8(C-10); 134.2(C-1”); 135.7(C-5); 151.5(C-3); 157.8(C-4”); 222.0(C-17). MS *m/z* (%): 405 (100, $[M+H]^+$).

*3-Methoxy-2-[(4-methoxyphenyl)ethyl]-13α-estra-1,3,5(10)-trien-17-one (**17**)*

As described in the general method, compound **9c** (50 mg, 0.12 mmol) was reacted with elemental hydrogen under catalytic conditions in an autoclave. Compound **17** was obtained as an oil (42 mg, 84%, R_f = 0.60). Anal. Calcd. for $C_{28}H_{34}O_3$: C, 80.35; H, 8.19. Found: C, 80.27; H, 8.29. ^{1}H NMR (500 MHz, $CDCl_3$) δ ppm : 1.06(s, 3H, 18-H_3); 2.80(overlapping multiplets, 6H, 6-H_2 and 2a-H_2 and 2b-H_2); 3.79 and 3.80(2xs, 2x3H, 3−OCH_3 and 4”−OCH_3); 6.55(s, 1H, 4-H); 6.83 and 7.13(2xd, 2x2H, *J* = 8.2 Hz, 2”-H, 3”-H, 5”-H, 6”-H); 6.99(s, 1H, 1-H); ^{13}C NMR δ ppm : 21.0; 25.1(C-18); 28.4(2C, C-2a and C-2b); 30.3; 32.1; 32.7; 33.5; 35.6; 41.4; 41.7; 49.3; 50.1(C-13); 55.3 and 55.4(4”−OCH_3 and 3−OCH_3); 110.5(C-4); 113.6 and 129.3(2x2C: C-2”, C-3”; C-5”, C-6”); 127.5(C-1); 127.9(C-2); 131.2(C-10); 134.7(C-1”); 135.3(C-5); 155.5(C-3); 157.7(C-4”); 221.6(C-17). MS *m/z* (%): 401 (53), 419 (100, $[M+H]^+$).

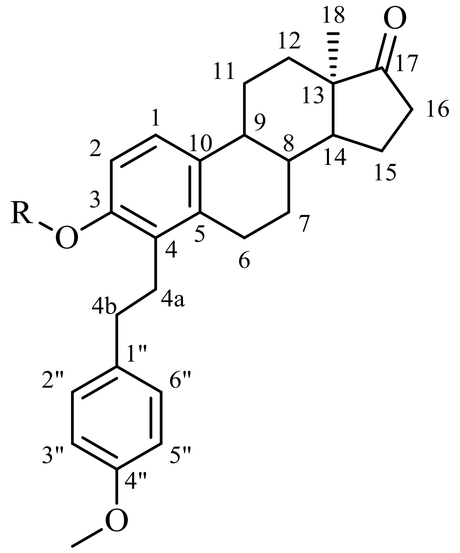

R = H, Me

*3-Hydroxy-4-[(4-methoxyphenyl)ethyl]-13α-estra-1,3,5(10)-trien-17-one (**18**)*

As described in the general method, compound **10c** (50 mg, 0.12 mmol) was reacted with elemental hydrogen under catalytic conditions in an autoclave. Compound **18** was obtained as a white solid (39 mg, 80%, Mp.: 157−159°C, R_f = 0.52). Anal. Calcd. for $C_{27}H_{32}O_3$: C, 80.16; H, 7.97 Found: C, 80.25; H, 8.05. ^{1}H NMR (500 MHz, $CDCl_3$) δ ppm : 1.06(s, 3H, 18-H_3); 2.63−2.85(overlapping multiplets, 6H, 6-H_2 and 4a-H_2 and 4b-H_2); 3.79(s, 3H, 4”−OCH_3); 4.65(s, 1H, OH); 6.61(d, 1H, *J* = 8.3 Hz, 2-H); 6.83 and 7.12(2xd, 2x2H, *J* = 8.3 Hz, 2”-H, 3”-H, 5”-H, 6”-H); 7.03(d, 1H, *J* = 8.3 Hz, 1-H); ^{13}C NMR δ ppm : 21.1; 25.1(C-18); 27.1; 28.5(2C, C-4a and C-4b); 28.6; 32.1; 33.5; 34.3; 40.8; 41.8; 49.3; 50.1(C-13); 55.3(4”−OCH_3); 112.9(C-2); 113.8 and 129.3(2x2C: C-2”, C-3”; C-5”, C-6”); 124.4(C-1); 125.7(C-4); 132.5(C-10); 134.4(C-1”); 136.4(C-5); 151.5(C-3); 157.8(C-4”); 221.9(C-17). MS *m/z* (%): 304 (100), 405 (72, $[M+H]^+$), 445 (94).

*3-Methoxy-4-[(4-methoxyphenyl)ethyl]-13α-estra-1,3,5(10)-trien-17-one (**19**)*

As described in the general method, compound **11c** (50 mg, 0.12 mmol) was reacted with elemental hydrogen under catalytic conditions in an autoclave. Compound **19** was obtained as an oil (43 mg, 86%, R_f = 0.62). Anal. Calcd. for $C_{28}H_{34}O_3$: C, 80.35; H, 8.19. Found: C, 80.22; H, 8.35. ^{1}H NMR (500 MHz, $CDCl_3$) δ ppm : 1.06(s, 3H, 18-H_3); 2.67 and 2.85(2x overlapping multiplets, 2x3H, 6-H_2 and 4a-H_2 and 4b-H_2); 3.80 and 3.81(2xs, 2x3H, 3−OCH_3 and 4”−OCH_3); 6.74(d, 1H, *J* = 8.5 Hz, 2-H); 6.84(d, 2H, *J*= 7.3 Hz, 3”-H, 5”-H); 7.14(overlapping multiplets, 3H, 1-H , 2”-H, 6”-H); ^{13}C NMR δ ppm : 21.1; 25.1(C-18); 27.1; 28.5; 28.6; 32.1; 33.5; 34.4; 40.8; 41.8; 49.3; 50.1(C-13); 55.3 and 55.4(4”−OCH_3 and 3−OCH_3); 108.1(C-2); 113.6 and 129.2(2x2C: C-2”, C-3”, C-5”, C-6”); 124.1(C-1); 127.9(C-4); 132.3(C-10); 134.9(C-1”); 136.0(C-5); 155.4(C-3); 157.7(C-4”); 221.7(C-17). MS *m/z* (%): 135 (100), 401 (91), 419 (97, $[M+H]^+$).

Determination of 17β-HSD1 inhibition

The inhibitory effects exerted on 17β-HSD1 activity by the newly synthesized conjugates were determined via an in vitro radiosubstrate incubation method described in detail earlier.[1]

[6,7-3H(N)]Estrone, S.A. = 50 Ci/mmol, was purchased from the American Radiolabeled Chemicals (St. Louis, MO, USA). Nonradioactive E1 and E2 standards, NADPH cofactor, other chemicals and solvents of analytical grade purity were purchased from Sigma (St. Louis, MO, USA) and Fluka (Buchs, Switzerland). Kieselgel-G TLC layers (Si 254 F, 0.25 mm thick) were obtained from Merck (Darmstadt, Germany). Human term placenta specimens were collected and used with the ethical approval of the Institutional Human Investigation Review Board. Human term placenta specimens were combined and homogenized with an Ultra-Turrax in 0.1 M HEPES buffer (pH 7.3) containing 1 mM EDTA and 1 mM dithiotreitol, and the cytosol was obtained by fractionated centrifugation. The substrate tritium-labeled E1 (1 µM, 250,000 dpm) was added to the incubate in 10 µl of 25 % v/v propylene glycol in HEPES buffer solution, whereas the test compounds were applied in 10 µl of dimethyl sulfoxide solution. (These organic solvent contents in the 200 µl final volume of the HEPES buffer incubation medium did not reduce the enzyme activity substantially.) Cofactor NADPH was used in excess concentration (100 µM). The enzymatic reaction was started by the addition of the cytosol aliquots. Incubation was carried out at 37 °C for 2.5 min and was then stopped by the addition of ethyl acetate and freezing. After extraction, unlabeled carriers of E1 and the product E2 were added to the samples. The two steroids were separated by TLC with the solvent system dichloromethane/diisopropyl ether/ethyl acetate (70:15:15 v/v) and UV spots were used to trace the separated steroids. Spots were cut out and the radioactivity of the E2 formed and of the E1 remaining was measured by means of liquid scintillation

counting. Test compounds were applied at 10 µM and their inhibitory effects were expressed by relative conversions, in comparison with the transformation measured in the noninhibited control incubation. Two measurements were performed for each test compound and the mean was calculated. The IC50 values (the inhibitor concentration which decreases the enzyme activity to 50%) were determined for the most effective test compounds. In these cases, conversions were measured at different concentrations in the interval 0.001–50 µM. IC50 results were calculated by using unweighted iterative least-squares logistic curve-fitting by means of the "absolute IC50 calculation" function of GraphPad Prism 4.0. The IC50 of unlabeled E1 was measured as reference and that was found to be 0.63 µM.

References

1 Bacsa, I.; Jójárt, R.; Schneider, G.; Wölfling, J.; Maróti, P.; Herman, B. E.; Szécsi, M.; Mernyák, E. *Steroids* **2015**, *104*, 230–6.

2 Szabó, J.; Jerkovics N.; Schneider G.; Wölfling J.; Bózsity N.; Minorics R.; Zupkó I.; Mernyák E. Molecules **2016**, *21*, 611
